# Selections and Abstracts

**Published:** 1915-12

**Authors:** 


### SELECTIONS AND ABSTRACTS

### THE FACTOR OF POVERTY IN SANITATION.

(EXCERPT FROM REMARKS OF SURGEON GENERAL GORGAS.)

The factor of poverty in sanitary problems was discussed in Washington, Nov. 26, by Surgeon General William C. Gorgas, whose success in cleaning up Havana and the Panama canal zone have brought him recognition as America's leading sanitarian. His audience was the Clinical Society of Surgeons, assembled in their twenty-fourth annual meeting. Dr. Gorgas said, in part:

"Such sanitary work as is necessary in the tropics is inexpensive, but measures directed against special disease are not the greatest good that can be accomplished by sanitation.

"Before these great results that we can all now see are possible for the sanitarian, we shall have to alleviate more or less the poverty at present existing in all civilized communities. Poverty is the greatest of all breeders of disease and the stonewall against which every sanitarian must finally impinge.

"During the last ten years of my sanitary work I have thought much on this subject. Of what practical measure could the modern sanitarian avail himself to alleviate the poverty of that class of our population which most needs sanitation? It is evident that this poverty is principally due to low wages; that low wages in modern communities are principally due to the fact that there are many more men competing for work than there are jobs to divide among these men. To alleviate this povety two methods are possible, either a measure directed toward decreasing the number of men competing for jobs, or, on the other hand, measures directed toward increasing the number of jobs.

"The modern sanitarian can very easily decrease the number of men competing for jobs: if by next summer he should introduce infected stegomyia mosquitos at a dozen different places in the southern United States he could practically guarantee that when winter came we would have several million less persons competing for jobs in the United States than we have at present. This has been the method that man has been subject to for the last six or seven thousand years, but it does not appeal to me, nor, I believe, to yourselves. This method is at present being tried on a huge scale by means of the great war in Europe. I do not think that I risk much in predicting that, when this war is over and we shall have eliminated three or four million of the most vigorous workers in Europe, wages will rise and for a long time no man will be unable anywhere in Europe to get a job at pretty fair wages.

"But I am sure that every sanitarian would much rather adopt measures looking toward the increase of jobs rather than, as we have done in the past, submit to measures that decrease the number of competitors for jobs.

"I recently heard one of the members of the Cabinet state that in the United States 55 per cent. of the arable land, for one reason or another, is being held out of use. Now suppose in the United States we could put into effect some measure that would force this 55 per cent. of our arable land into use. The effect at once would be to double the number of jobs. If the jobs were doubled in number, wages would be doubly increased. The only way I can think of forcing this unused land into use is a tax on land values.

"I therefore urge for your consideration, as the most important sanitary measure that can be at present devised, a tax on land values."

### IT IS ESTIMATED THAT 50 PER CENT. OF ALL BLINDNESS IS PREVENTABLE.

This statement will be surprising to many—that one-half of the sightless people in this country need not have been blind had proper care been given to their eyes. But it has long been known by those endeavoring to prevent unnecessary blindness that more than a quarter of the pupils in the schools for the blind are sightless because their eyes were not properly treated during the first few days of life; that poor midwives are in part responsible for this tragedy; that children become totally or partially blind from neglected "sore" and "weak" eyes, and from neglect after attacks of such infectious diseases as measles, scarlet fever, etc.; that progressive nearsightedness among children may cause total or partial blindness if neglected; that household and industrial accidents cause the loss of many eyes; that drinking wood alcohol or inhaling its fumes in close places causes both blindness and death; that inadequate lighting and glaring surfaces are responsible for much visual disturbance, including eye-strain; and that eye-strain is a frequent cause of both mental and physical inefficiency.

"Babies' Sore Eyes" (ophthalmia neonatorum)

This disease, which causes so much blindness, is preventable and, if taken in time, is curable.

The prevention of blindness from babies' sore eyes is accomplished through the routine use of 1 per cent. solution of silver nitrate, or some such prophylactic, in all infants' eyes immediately after birth, and by prompt and skilful treatment of babies' eyes when they become red, swollen and discharging, whether or not a prophylactic has been used.

1. Does the birth certificate used in your locality include the question, "What preventive did you use for ophthalmia neonatorum? If none, state the reason therefor"?
2. Are prophylactic outfits distributed gratuitously by your Health Officer to doctors and midwives?

3. Are doctors, midwives and parents required to report to the Health Officer, within six hours, redness, swelling or discharge from the eyes of infants in their care who are under three weeks of age?
4. Is this reporting law printed on the birth certificate —thus acting as a constant reminder?
5. Has the Department of Health a nurse in its employ, or does it so co-operate with a nursing organization that it may send a nurse at once to visit each reported case and secure adequate medical or hospital treatment for uncared-for cases?
6. Are there such hospital facilities for the care of babies' sore eyes that the Health Officer may send an infant to a hospital without delay if the eyes are in a serious condition?

Take these points up with your Health Officer, interested oculists and obstetricians, and don't rest until they are all attended to. Make it your business to see that any baby suffering from sore eyes, of which you have knowledge, is given prompt and adequate medical attention.

Try to have at least one nurse in the communtiy for eye work exclusively, and see that there are hospital facilities for treatment of severe cases of babies' sore eyes.

### Midwives.

These women attend about half the births occurring in this country, and the majority of them are dirty, ignorant and generally unfit to assume the care of mothers and babies. Although the carelessness of many physicians is equally reprehensible, it is due in great measure to the ignorance and neglect on the part of midwives that many babies become blind from babies' sore eyes.

1. Are there midwives practising in your community?
2. Are they registered by an offical body?
3. Is it required that they be adequately trained; pass an examination; obtain a license; and register before beginning to practise?

4. Has your community a midwife training school connected with a good hospital?
5. Do the practising midwives give clean, careful nursing care to mother and child, and instruction to the mother concerning hygiene of pregnancy and care of her child?
6. Has the State or City Health Department adopted rules regulating midwives' practise in detail and requiring them to summon a physician in all but normal cases?
7. Are there inspectors to enforce the rules and give helpful advice to the midwives?

Make it your business to find out about this, for the sake of the mothers and babies. Your Board of Health is the proper body to have control of midwives. The Board of Education should regulate their training and licensure.

EYESIGHT OF SCHOOL-CHILDREN.

Many normal children seem backward because they have sore eyes or defective vision. Failure to correct these defects will probably mean continued retardation for many of the children, and inability to reach their highest possible mental and physical development and economic efficiency. Continued neglect may result in partial or total blindness.

1. Are all class-rooms in your school adequately lighted?
2. Are the blackboards and tops of the desks lusterless?
3. Are all the desks adjustable?
4. Are the children's eyes carefully and regularly examined for nearsightedness and other visual defects, and for various kinds of "sore" eyes?
5. Is this done by an oculist?
6. Are there clinics where school-children with "sore" or "weak" eyes may be treated?
7. Is there provision for furnishing eye-glasses to indigent children who need them?
8. Are common towels allowed in your schools? (They spread eye diseases.)

9. Are the children taught how to take care of their eyes?

Improving the eyesight and general surroundings of school-children will be of immediate benefit to them, and will increase their chances for enjoying health and prosperity later in life.

Talk to your Board of Education about this—it is important. The children can't do it themselves.

### Industrial Accidents.

Many good workmen are seriously handicapped and even become public charges as a result of losing one or both eyes in an accident that might have been prevented. Men, women and children often suffer from severe eye-strain because they are not provided with adequate light while at work.

1. Are workmen in the factories and shops in your locality protected from eye accidents by goggles; guards on emory wheels; screens to catch flying chips; guards on water gauges; etc.?
2. Are the factories, workshops and workroom adequately lighted?
3. Are workmen examined to see that they are not especially liable to accidents because of defective vision?

Take these points up with your Department of Labor, Industrial Safety Commission, or some similar body.

The eyes are bread-winners and must be carefully guarded.

### Wood Alcohol.

Wood alcohol is a poison which may cause blindness or death if swallowed, or if its fumes are inhaled in an inadequately ventilated place.

1. Have you a law forbidding wood alcohol to be sold in any form without a poison label and warning?
2. Is the use of wood alcohol absolutely forbidden in beverages, medicines and toilet preparations?

3. Are your druggists, paint and varnish dealers, liquor dealers, grocers and barbers prosecuted for failure to comply with the above restrictions?
4. Is wood alcohol used in any of your local industries? If so, are employers required to protect their workmen from poisoning by providing adequate ventilation?

The National Committee for the Prevention of Blindness wants your help and co-operation in spreading the knowledge that much blindness is needless. It has data and information, lantern slides, exhibits and pamphlets on the various causes of unnecessary blindness and methods of prevention, and it is glad to share these with workers in all parts of the country.

In order to accomplish the ends suggested in the foregoing program, it is necessary to have official action, supported by public opinion. Try to have at least one big popular meeting annually under the joint auspices of the local Medical Society, the Health Officer, Superintendent of Schools, Y. M. C. A., women's clubs, nursing organizations and relief agencies. Arrange for talks before school children, mothers' clubs, etc., and secure as much newspaper publicity as possible. Write to the National Committee for suggestions and assistance.

THE EDUCATIONAL WORK MUST BE SUSTAINED—THE EFFORT UNREMITTING.

ELLA L. BLAIR,
Chairman, Public Health Department, General Federation of Women's Clubs.

CAROLYN C. VAN BLARCOM,
Chairman, Committee on Prevention of Blindness and Midwives, National Organization for Public Health Nursing.
Secretary, National Committee for the Prevention of Blindness, 130 East 22nd Street, New York City.

### THE DOG AS A CARRIER OF DISEASE TO STOCK.

The dog in the country is a useful and pleasant adjunct to the farm if he is properly controlled and cared for, but when neglected, may readily become a carrier of disease to stock, in addition to gaining opportunity to kill sheep and destroy gardens and other property. Dog ordinances, as a general rule, have been intended chiefly to curb the dog's power of doing harm by attacking, biting, killing or running sheep or stock. The part that he plays as a carrier of diseases to animals only recently has been recognized, according to the zoologists of the Department of Agriculture, who believe that when this is better understood, rural ordinances and laws which lessen this danger will gain the support of the community.

Of the diseases carried to stock by dogs, the foot-and-mouth diseases is probably of the greatest interest at this time. In this case the dog acts as a mechanical carrier of infection. The dog which runs across an infected farm easily may carry in the dirt on his feet the virus of this most contagious of animal diseases to other farms and thus spread the disease to the neighboring herds. In infected localities it is absolutely essential, therefore, to keep all dogs chained and never to allow them off the farm except on leash.

There are, however, many other maladies in the spread of which the dog takes an active part. In Bulletin 260 of the United States Department of Agriculture, "The Dog as a Carrier of Parasites and Disease," it is pointed out that rabies, hydatid, ringworm, favus, double-pored tapeworm, and tongue-worm are often conveyed to human beings in this way. It occasionally happens also that the dog helps fleas and ticks in transmitting bubonic plague or the deadly spotted fever.

Hydatid disease is caused by the presence in the liver, kidneys, brain, lungs, and other organs, of a bladder worm or larval tapeworm. Bladder worms are often as large as an orange and may be larger. A dog which is allowed to feed on carrion or the raw viscera of slaughtered animals may eat all or part of a bladder worm containing numerous tapeworm heads. These tapeworm heads develop into small segmented tapeworms in the intestines of the dog. The tapeworms in turn develop eggs which are passed out in the excrement of the dog. They are spread broadcast on grass and in drinking water where animals can very well eat them and thus become infected The hog is particularly liable to this disease because of its rooting habits. The eggs may get into human food, and persons who allow dogs to lick their hands and face also run the risk of getting the eggs of the tapeworm in their systems.

Prevention on the farm consists in so restraining the dog that he can not get at carrion or raw viscera. Viscera should be boiled before being fed to dogs and should never be thrown on the fields. If not cooked and fed, viscera and carcasses should be burned, buried with lime, or so disposed of as not to be accessible to dogs. Proper feeding of the dog is essential, and the owner who does not feed a dog properly has no right to keep one.

The parasite which causes gid in sheep somewhat resembles the hydatid worm. A dog allowed to eat the brain of a giddy sheep may swallow this parasite and later distribute the eggs of the resulting tapeworm over the pasture. Sheep while grazing swallow the eggs with the grass which they eat. In the case of sheep dogs it is important to administer vermifuges often enough to keep them free of these worms. In the case of sheep measles, the bladder worm in the meat, typical of this disease, is swallowed by the dog and again the tapeworm eggs are passed by the dog to grass or water, and there are eaten by sheep.

Of the external parasites which dogs may carry to animals, fleas and the various kinds of ticks are both troublesome and dangerous. The remedy is clear. The owner must keep his dog clean, not merely for the comfort and happiness of the dog, but to prevent it from becoming a carrier of disagreeable and dangerous vermin.

These reasonable measures, important to the stock of the farm, have a direct connection with the health of the family.

Where ringworm or other skin diseases break out among the children, or the worm parasites develop, it is well to determine whether a dirty or uncared-for dog may not be carrying infection on his skin or hair, or be conveying disease from carrion directly to the food and persons of his friends. Even if no one is infected with the disease, the folly of allowing a dog to remain dirty and have the freedom of a home where personal cleanliness and hygiene are respected, is apparent.—Office of Information, U. S. Department of Agriculture.

---

### THREE PSEUDONYMS OF EARLY TUBERCULOSIS.

F. M. Smith, M. D., Abingdon, Va.

The art of diagnosis is akin to the detective's, and there is a sort of fascination in following out the analogy. It gives to work the zest of a game, this matching of wits against disease in the role of the criminal. The diagnostician gets his clue from the complaint of the patient. Headache, pain, fever, weakness, nervousness, indigestion, cough, etc., are naked manifestations of the disease; some offense against the peace and dignity of the healthful body. What is the crime and what the criminal agent? The answer may tax all of the resources of our diagnostic machinery.

As in criminology the bare facts of crime suggest its full particulars, so in medicine the complaint of the patient should suggest disease. As a detective segregates a group of criminals, one of which is probably guilty of the particular crime, so we should be able to form in our minds a group of diseases forcefully suggested by the complaint. Then follows the history and examination, like the ramified investigations of the police department, by which our attention is centered upon some one culprit, which we now proceed to bring to trial by due process of diagnostic laws. Jumping at conclusions, the making of diagnoses upon surface impressions is just as culpable in the practice of medicine as mob-law would be in criminal practice. We must try our cases by the best rules of evidence at our disposal, to convict the true criminal, and none other, of the full extent of his crime.

We are often as faulty in our diagnosis as would be the police department which, when looking for a criminal named "John Smith," would release a suspect merely because he himself says that his name is not John Smith but "Sam Brown." The assuming of an alias is as common in medicine as in criminology, and just as apt to mislead: we take the patient's name for his illness and are satisfied.

This introduces us to our subject: Three Pseudonyms, or, if you please, Aliases, of Early Pulmonary Tuberculosis.

Within a certain term of years, we have made 157 diagnoses of pulmonary tuberculosis. Upon reviewing them, it is probable that 7 of them would be excluded to-day, the evidence being insufficient. Of the 150 remaining, the complaint had nothing to do with the pulmonary condition in 18 who consulted us because of a more prominent tuberculous condition elsewhere. If one has chronic bladder disturbance with pyuria and hematuria, enlargements of glands, a chronic osteomyelitis, or other extra-pulmonary condition which proves tuberculous, it is a safe guess that most of such individuals have the same process in the lungs. So with these 18.

Of the 132 now remaining, the complaint which brought the patients for examination was:

| | |
|---|---|
| Bronchial or Catarrhal Trouble in | 49 |
| Stomach Trouble in | 29 |
| Weakness, Nervousness, or both, in | 35 |
| Indigestion and Nervousness in | 6 |
| Headache, Backache, or both, in | 9 |
| Miscellaneous Complaint in | 4 |

We are liable to be led into error just here by the fact that a number of tuberculous patients have some complicating illness. For instance, appendicitis is often a complication. In such a case, to which should be attributed the patient's complaint of stomach trouble?

In order to be fair, though at considerable sacrifice, the cases have been subjected to critical survey, with the result that we find 89 uncomplicated cases of tuberculosis in which the illness has been called:

Bronchial or Catarrhal Trouble in ------48%
Indigestion in ----------------------------21%
Nervous Prostration in -----------------25%

Bronchial Catarrh, Indigestion and Nervous Prostration, then, are the three pseudonyms, or aliases, of pulmonary tuberculosis to which reference is made.

It may seem that the percentages above are a reflection on the profession of the community from which most of these patients come. But the figures should not be read at their full face value for such indictment. In many the physician referring the patient was fully cognizant of the true and complete diagnosis, in which case the patient was referred merely for confirmation.

Yet many of the profession still, like many laymen, have a false conception of the typical picture of the early tuberculous patient. They have a conception which has not changed since Charles Dickens wrote into Nicholas Nickleby: "There is a dread disease * * * in which death and life are so strangely blended that death takes on the glow and hue of life, and life the gaunt and grisled form of death; a disease which medicine never cured, wealth never warded off, or poverty could boast exemption from; which sometimes moves in giant strides, and sometimes at a tardy, sluggish pace, but slow or quick, is ever sure and certain." So, many doctors still think of tuberculosis only in terms of consumption. And yet tuberculosis is just as different from consumption as a boil is from general septicemia. When the profession generally can think of a "boil," as it were, segregated in one corner of the lung, before it becomes a diffuse infection, with cavitation perhaps, all through the lungs, then is caught the distinction which is sought.

Such outstanding symptoms as cough with expectoration, chest pain, dyspnea, hoarseness, even hemoptysis, have been referred under the diagnosis of bronchial catarrh, recurrent attacks of grip, asthma, intercostal neuralgia, etc. Even today there are numbers of the profession who will not accept a diagnosis of tuberculosis until bacilli can be demonstrated in the sputum.

Of the many causes of indigestion, pulmonary tuberculosis is one of the most frequent. In Dr. Cabot's work it was found the second most frequent cause. As he says, the vast majority of the causes of indigestion have nothing to do with the stomach at all, that is with any disease of the stomach. It, on the other hand, bears the brunt of any abnormality in any other organ. It behaves as if it were the barometer of the body: any storm brewing, even though still distant from the stomach, disturbs its normal equilibrium. "The truly gastric causes of indigestion may be reduced almost entirely to two, cancer and ulcer. Nervous dyspepsia is fearfully common, but it does not originate in the stomach." This is a point which we should constantly bear in mind. It is a pitifully inadequate diagnosis to tell the patient whose indigestion originates in the toxemia of tuberculosis that he has nervous dyspepsia. And yet this has been the belief of 21 per cent. of our patients. Gastric complaints are all the patient had. Yet "unexplained indigestion coming on in a person habitually healthy, in a person who has not changed his diet or his work, who is not anemic or nephritic, or overwhelmed by mental torture or worry, should be suspected of beng due to tuberculosis. True gastric indigestion should have a demonstrable cause, either local in the stomach itself (cancer or ulcer), or an external cause in some obvious indiscretion of diet. We often badger the patient into a forced and reluctant admission of such indiscretion, when in truth there has been none."

The neurasthenic group of tuberculous patients is the most interesting of all. Whenever a person under middle age complains of weakness and nervousness, of headache, mental lassitude, irritability and fatiguability, especially if there is also pallor, we should suspect tuberculosis. After 40, it is true, we have also to remember arteriosclerotic changes of the kidneys and alsewhere. But the point is that typical neurasthenic symptoms mean true neurasthenia even more rarely than indigestion means stomach trouble. A complaint of nervousness and weakness rarely indicates true nervous disease, but oftenest a demonstrable toxemia or infection. A diagnosis of nervous prostration on my own records stands as an admission of inability to make a diagnosis.

It is tempting to follow these rather iconoclastic remarks with something more constructive; to take up systematically the clinical diagnosis of early pulmonary tuberculosis. But this is not the point to emphasize. Surely the signs and symptoms of this condition are common knowledge or are readily available. In fact, there should be no more hesitancy in arriving at a positive diagnosis than in any other disease. The trouble is, oftenest, in not embracing it in the probabilities of the individual case, and, therefore, in not searching for its signs.

Whenever a patient complains of bronchial trouble, of indigestion, or of neurasthenic symptoms, we should seize at once upon the significance of these complaints as sign-posts on the diagnostic road. Careful questioning as to recent or remote exposure in the family as elsewhere should follow. Confirmatory signs of loss in weight, an inability to do easily and protractedly his ordinary work; an unexplained loss of appetite; and the symptoms in general of the "run-down" condition, should prompt the course of taking the patient's temperature at least morning and night for a week.

Fever is the most characteristic sign of an active tuberculosis. Yet fever alone is not diagnostic. It simply means infection. This should be emphasized, because syphilis, malaria and cryptic pyogenic infection may all have confusing points of resemblance. Characteristic of the fever of early tuberculosis is (1) its chronicity: the same range day after day, so long as the patient lives his routine life; (2) its low range: we expect a low fever in the early case, somewhere in the neighborhood of 100 degrees; (3) its intermittent character: subnormal temperature in the mornings is almost as significant as a rise to 99.5 degrees or 100 degrees in the afternoon; (4) the mild constitutional reaction: the patient is usually unaware that he has any fever; (5) the fever disappears when the patient is put to bed for a few days, and is apt to rise again if he gets up too soon.

However much we may question the absolute diagnostic significance of any one or all of these several points, of one point we may be certain: The patient who is running fever of this sort, and whose pulse is disproportionately quick, or excitable, has neither simple bronchial catarrh, indigestion or nervous prostration. Any one of these diagnoses becomes now nonsensical.

A lung examination should be made with the utmost care in a quiet room. Whether or not satisfying sounds are to be heard depends mostly upon the examiner: :not only upon whether he is expert enough to elicit the signs, but whether he is willing to recognize them when he hears them. For the signs in truly early cases are limited in distribution, are few and small. Often they do not come out with ordinary breathing or even deep breathing. The patient must be made to cough after a forced expiration and before a forced inspiration, to get the finest crackling rale the prettiest sign of all, and pathognomonic if any one sign is.

Yet, together with many other, I believe more correct diagnoses will be made, and the greatest good to the largest number will accrue, if the stethoscope is less relied on and the thermometer more so. Every doctor should strive, surely, to perfect himself in the use of the stethoscope, but while striving after perfection he should not ignore the other evidence becasue he fails to find the rale or some other sign.

If ninety-four per cent. of early tuberculosis cases complain of bronchial catarrh, or of dyspepsia or of nervous prostration, the conclusion is obvious: We should assume every such case to be possibly tuberculosis; and we have no right to give any such false name to any such case until we are satisfied by the most thorough study that he is not such. Haphazard diagnoses no longer pay: there are too many others doing good work. There is no disease in which superficial appearances are more deceiving and in which conscientious study is more enlightening than tuberculosis. It is astonishing, also, how many will get well of their catarrhal symptoms, of dyspepsia, and of neurasthenia on rational treatment directed entirely to the tuberculosis process—The Old Dominion Journal of Medicine and Surgery, November, 1915.

Abingdon Hospital.

---

### SUMMARY OF THE ANNUAL REPORT OF THE SURGEON GENERAL OF THE UNITED STATES PUBLIC HEALH SERVICE.

The annual report of the surgeon general of the United States Public Health Service records the largest amount of work performed in the history of that organization. Since the passage of the law of 1912 the public health functions of the service have materially broadened, thereby increasing greatly its usefulness to the American people. Throughout the report the economic importance of disease prevention is made apparent to the reader.

Perhaps the most important achievement of the year was the discovery that pellagra is a deprivation disease, resulting from a faulty diet containing an excess of carbo-hydrates. While the final experiments which led to this discovery have only recently been completed, the conclusion itself is the culmination of investigations extending over a period of seven years. The work has consisted of epidemiological field studies, actual feeding experiments conducted at numerous places in Georgia and Mississippi, and experimental research at Spartanburg, South Carolina, and other places.

The new national quarantine station was opened at Galveston, Texas, and the control of the Boston station was transferred to the Public Health Service. A great reduction in immigration has resulted during the year, with a corresponding increase in the number of aliens certified. At the port of New York, the percentage has risen from 2.29, previous to the development of the European conflict, to 5.37 since that time; this increase largely being due to the fact that with the decreased immigration more time can be devoted to the examination. The number of cases treated at Marine Hospitals and relief stations exceeded 55,000, 15,000 of which were hospital patients, a considerable increase over previous years. The coast guard cutter "Androscoggin" was fitted out as a hospital ship and now affords relief to deep sea fishermen on the banks of Newfoundland.

On the occurrence of plague at New Orleans, the first outbreak upon the Gulf seaboard, the state and local health authorities requested the Public Health Service to take charge of the situation. Extensive rat-proofing and other anti-plague measures were undertaken, resulting in the eradication of the disease from among human beings, and the practical extermination of the rodent infection.

Great reduction in the incidence of malaria was obtained in localities where surveys were conducted. Drainage projects, rice culture studies and the conditions surrounding the impounding of water for power purposes were investigated in order to eradicate as far as possible the disease in these areas. Scientific investigations of malarial infection showed that in the latitude of this country the most important agent in carrying the infection through the winter season is man, and not the infected, hibernating, Anopheles mosquitoes as was previously supposed. From the standpoint of prevention this is a discovery of considerable value.

Studies of occupational diseases and industrial hygiene were instituted at several places during the year. A survey of the industries of Cincinnati was made to determine the cause of the prevalence of tuberculosis among industrial workers. The investigations relating to the migration of persons suffering from tuberculosis were completed.

Upon the request of the health authorities of five states, the organization and operations of the respective boards of health were studied and recommendations advanced for improvement in the powers and duties of these bodies. The health organizations of several cities were likewise investigated.

Investigations of the pollution of streams and the examination of shellfish were also conducted.

Trachoma was combated in the Appalachian Mountains, where it is most prevalent, over 12,000 cases being treated. Surveys in certain states during the year showed that the disease is not an uncommon infection.

Rural sanitation work was conducted in six different states and everywhere resulted in the reduction of typhoid and other communicable diseases.

Public health laboratories for the prevention of the interstate spread of disease were established at Chicago, Seattle, and numerous other railway centers.

Additional duties have been imposed upon the service by extension of relief benefits to the newly organized Coast Guard and the physical examination of seamen applying for the rating of "able seaman." For this reason, and because of the greatly increased health functions of the service, an increase in the commissioned personnel is recommended. An additional building for the Hygiene Laboratory and the establishment of a National Leprosarium for the proper segregation and care of cases of leprosy are also recommended.

---

### SAFETY FIRST FOR CHILDREN.

### Important Book Just Issued for Conservation of Human Life.

### Contains Charming Story Giving Powerful Object Lesson of Caution to City Children.

### Produced by Safety First Federation of America for General Public Education and for Especial Use in Public Schools.

"Safety First for Children" is the title of a little book just issued by the Safety First Federation of America. It is a neat little thing in itself but has behind it a most ambitious purpose, the conservation of human life. Very large effort has been expended in its preparation and it seems destined to have widespread influence throughout the United States.

There is an interesting bit of history connected with its authorship. The Safety First Movement is a familiar thing. It began several years ago and spread like wildfire throughout the country. It had its origin in enlightened selfishness, which is the very essence of humanitarianism. The great traffic and industrial corporations were striving to minimizez the number of violent accidents and eliminate the destruction of property due to the carelessness of their own employes and the public in general. This idea was enlarged upon by interested workers and it developed into a general movement for the public welfare. Safety First societies were established in the important cities of the United States.

### A National Safety First Movement.

The next logical step in the movement was the co-operation of the work of these various societies through the formation of a Safety First Federation. This is made up of representatives of local Safety First societies and city officials from all parts of the United States, and with headquarters in New York City. This Federation has for its object the conservation of life and property in the United States. It is conducting its work on two broad general lines: first, by regulation, and second, through education.

The work of regulation is being accomplished in the study of the causes of preventable accidents and securing the passage of state and federal laws and city ordinances that will regulate traffic and industry so as to avoid them.

### A Matter of Education.

Laws can regulate what people do to or with each other but they can not regulate what people will do with or to themselves. The society therefore realized early in the movement that it was necessary to educate the people for their own self-protection—that modern conditions made necessary the training of people in habits of caution and care. Very little can be accomplished with grown people because the adult mind and body are both fixed in their habits. The Society therefore was convinced that their efforts should be centered upon the education of children.

Officials in numerous cities were desirous of introducing some form of instruction in the public schools and immediately a demand was made upon the officials of the Safety First Federation for a text book which could be used for this purpose.

### Gathering the Materials.

Mr. Frederick H. Elliott, the Executive Secretary of the Federation, immediately set about the task of preparing the necessary work. His materials were gathered from various sources. Police and traffic officials in numerous cities, lecturers and instructors for industrial corporations who were conducting educational courses, school teachers and school officials who had made a special study of child psychology were called upon for their experiences, opinions and suggestions.

A peculiar conflict of opinion was immediately disclosed. The purpose of the book was to warn children against accidents. Accidents are in themselves violent and contain an element of horror. They also, if shown, would constitute the things which children were to be asked "not to do." All this was in direct conflict with the advanced theory of modern teaching. Instructors to-day say that nothing hideous or terrifying should be presented to the child mind, and, moreover, that negative instruction of any character is worse than useless, merely serving to confuse the child and direct his attention to the things from which it was desired to divert him. The police officials and city officials generally were of the opinion that a vigorous and rigorous course of instruction on "What not to do," however, should be given.

### Conflict of Opinion.

The school officials were positive that anything in the nature of a text book with tedious rules to be learned would be distasteful to children and would defeat the very purpose of the course. Traffic officials were convinced that it was necessary to present a series of "don'ts" and brought to their defense the fact that the Ten Commandments, upon which the modern Christian morality was based, consist primarily of prohibitions. The problem was solved in a rather simple and ingenious manner by embodying in the book the story of the experiences of two boys in a modern city. Narrative is conceded to be the most powerful means of conveying instruction and the example of what someone else has done seems to have more weight with young people than any amount of admonition from the older folks.

### Education by Example.

The story tells of George Benton's visit to his cousin Arthur, who lives in the city. George is a country boy, unfamiliar with the ways of the city. Through nine chapters one follows his experiences and observations in avoiding the things which beset the paths of dwellers in the city and he is so profoundly impressed with what he goes through that he is impelled to do something to help other children out of similar difficulties. He conceives an idea, which he conceals from his cousin Arthur, but which is shown to be developing through the progress of an entire story and as it develops Arthur's curiosity continues to increase.

The great idea is finally disclosed in the last chapter, where George confides to Arthur his plan of compiling a set of Safety First rules for children, and together they enter into the work. Their first draft is submitted to Arthur's father, who heartily commends the idea and makes suggestions and addition which the boys had overlooked. The list as finally revised and adopted by the boys is given at the end of the book.

### Fire Prevention Chapter.

Supplementing this general story and set of rules is a chapter on fire prevention by Robert Adamson, Fire Commissioner of the City of New York, in which is included a series of photographs showing a number of the principal causes of fires in the home.

There are also some jingles of the "Mother Goose" order and a few verses or adages or memory gems with the Safety First flavor. All these being for the purpose of adding interest, variety and attractiveness to the general subject.

The book is attractively printed in four colors and with an illustration on very nearly every page. It is bound in boards with paper cover of a serviceable color, intended primarily for school use.

FOR GENERAL SCHOOL USE.

It is the purpose of the Federation to have the book introduced generally into the public schools of the United States, but until this is accomplished, a wide distribution will be secured through direct sales to the public. "Safety First for Children" has been named "the best gift book of the season," for the reason that in itself it is an attractive and interesting story book for children, and further, because it teaches a vital lesson in the conservation of human life.

During the present holiday season the book will be on display in practically all of the city department stores and book stores throughout the United States. It is also being considered by school boards in numerous cities, and in all probability "Safety First for Children" will attain a distribution which will make the famous figures of "Uncle Tom's Cabin" and the "Message to Garcia" sink into insignificance.

---

### PRISON AND PRUDERY VS. PURITY.

By G. HENRI BOGART, M. D., Shelbyville, Ill.

William Sanger was tried and found guilty the other day by three New York judges on the charge of circulating "obscene" literature. As the presiding judge sentenced him, he added insult to the sentence by saying, "The sooner society gets rid of the like of you the better."

The obscene literature which Sanger circulated was the little book written by his wife, Margaret Sanger, entitled "Family

Limitation," giving scientific information as to how conception may be prevented by clean, sane and wholesome methods, and the function of reproduction controlled. It was written primarily to circulate among the poor classes of New York, and is based upon the ethical and scientific principle that indiscriminate and excessive breeding is a menace, an immoral practice.

The arrest was procured by a trick. An agent of the Comstock society went to Mr. Sanger with a pitiful tale of conditions, claimed to have been befriended by Mrs. Sanger, and asked a copy of her book. As a matter of course it was secured and then made the basis of the indictment.

Twice in court the defendant attempted to secure a trial by jury but was denied, and was finally tried by a bench of judges.

In this hearing Sanger said: "It is the law, and not I, that is on trial here today." If this pamphlet on 'Family Limitation' is considered obscene and indecent, then all the medical books on the subject are also obscene and indecent."

In his final defense Sanger said: "The race has long ago emerged from the era of witchcraft, but yet today witchcraft exists in a different form, in the shape of obscenity laws. Obscenity statutes cannot be regarded in the same light as statutes dealing with concrete offenses, such as criminal libel, arson, or the like, because they are based on a state of mind. Things obscene can only be detected when we think in terms of deformed mental process of reasoning. Obscenity, like witches, will cease to exist when men cease to believe in it.

"The obscenity statute is therefore based on a belief rooted in the meanest, most cruel and ignorant censorship since the days of witchcraft. Shall any man or woman be liable to a fine or imprisonment based on belief more or less religious and on the attitude of mind of those mentally deformed?

"It can be shown that the framers of this obscenity statute knew nothing of the untold hardships endured by the workers in the past because information in regard to birth control was withheld from them. They were indifferent to or sublimely ignorant of the fact that the statute, with others of the same character, were and are the message of death to the workers of this generation. It was a white-livered morality they had in mind and not the crying need of humanity.

"The result of the enforcement of the law is that it has caused untold hardships to that stratum of society least able to help itself. All because of this obscenity law and others, the relief asked for by the workers is denied them and the giving out of information on birth control is made a prison offense. But the workers keep on having children without any means for their support. The breeding and not the rearing, of children goes on. These children come into the world not really wanted, because there is no place for them. They are shoved out in the streets, collect in gangs, are swatted like flies in places they call homes, largely because of these infamous obscenity laws. The homes of the very poor have been made brothels, instead of havens for the rising generation, where these children might have a chance to grow up in beauty and love, basking in the sunshine of their parentage. It has been shown that among families earning $500 a year one out of every four dies before he reaches one year of age. How about the unemployed? Their children become destitute and are thrown into orphan asylums and the like. If birth control information was not declared obscene, indecent, lascivious and disgusting, fewer and better children would be born and fewer children would die."

Margearet Sanger is under indictment for writing the book. She is now in London, England, but will return at once to stand trial. She says that the very publicity or unjust trial and punishment will do more to propagate her work than any other possible event, and she believes so completely in the righteousness of her cause that she offers herself as a willing sacrifice.

So far the sleuths have been unable to trace any of her books outside of New York State, which renders the infamous Federal law inoperative against her. So the proceedings have been brought under the laws of New York, whose court declares that it is a wrong worse than murder to teach the women of the submerged how intelligently to control their bodies, and to prevent the hideous wrong of bringing children into the slums to grown into vice, poverty, misery that is beyond the comprehension of those whose lives have been spent under God's great out-of-doors, under the glow of sunshine, where birds are heard.

"This community, like many others, suffers from a lack of children," said the judge who pronounced sentence upon Sanger. "The trouble is that many women in it are too selfish. I think that a lot of those who are devoting their time to equal suffrage, as Christian women, ought to go around advocating child-birth. It would be better for the community."

"According to this judge, it is "Christian" and for the good of the community to bear children to grow up in poverty, misery and ignorance; to become the victims of vice and crime and the sodden slaves of the masters of industry; and when these masters have profits to be defended somewhere, to go to war and be sacrificed on the crimson altar of greed.

The world may never progress to better, purer, higher things by looking backward, we must look forward and upward, for betterment of the race.—The Medical Fortnightly and Laboratory News.

